# Measuring citizens’ engagement during emergencies: Psychometric validation of the Public Health Engagement Scale for Emergency Settings (PHEs-E)

**DOI:** 10.1371/journal.pone.0261733

**Published:** 2021-12-22

**Authors:** Guendalina Graffigna, Lorenzo Palamenghi, Serena Barello, Mariarosaria Savarese, Greta Castellini, Edoardo Lozza, Andrea Bonanomi

**Affiliations:** 1 Engageminds HUB – Consumer, Food & Health Engagement Research Center, Università Cattolica del Sacro Cuore, Milan, Italy; 2 Department of Psychology, Università Cattolica del Sacro Cuore, Milan, Italy; 3 Faculty of Agricultural, Nutrition and Environmental Sciences, Università Cattolica del Sacro Cuore, Milan, Italy; 4 Department of Statistical Science, Università Cattolica del Sacro Cuore, Milan, Italy; Fondazione Istituto Neurologico Nazionale C Mondino Istituto di Ricovero e Cura a Carattere Scientifico, ITALY

## Abstract

The Covid-19 pandemic has highlighted the importance of citizens’ behaviors in the containment of the virus. Individuals might change their intention to adhere to public health prescriptions depending on various personal characteristics, including their own emotional status, which has been recognized to be a crucial psychological factor in orienting people’s adherence to public health recommendation during emergency settings. In particular, it is crucial to support citizens’ alliance with authorities and feeling of trust: public engagement is a concept that refers to the general involvement of citizens into public affairs which is generally considered an effective approach to enhance citizens’ understanding of their crucial role in public affairs. However, so far there is no agreement on the metrics and indexes that should be used to measures public engagement during a health crisis. The aim of this paper is to validate a psychometric scale (PHEs-E), which intends to measure the readiness of individuals to adhere to the prescribed behavioral change to contain the emergency. Data were collected throughout the pandemic in Italy: in particular, five independent samples were recruited starting from March 2020 to March 2021. Results showed that the proposed measure has good psychometric characteristics. A general linear model was computed to assess the differences of public engagement across the different data points and among citizens with different sociodemographic characteristics. Correlations with other psychological constructs (i.e. Anxiety, Depression and Self-Efficacy) were also tested, showing that more engaged citizens have a lower level of anxiety and depression, and a higher self-efficacy. This study’s findings indicate that individuals’ characteristics may differentiate citizens’ motivation to engage in public health behavioral recommendation to prevent the COVID-19 contagion. However the scale could be useful to perform a psychological monitoring of psychological readiness to engage in public health strategies to face critical events and settings.

## Introduction

The Covid-19 pandemic has undoubtedly underlined the relevance of citizens’ behaviors in containing or spreading the contagions [1]. In this situation, an important effort has been made worldwide in providing communication initiatives aimed at motivating the public to adhere to the recommended preventive measures such as wearing face masks, cleaning hands, and maintaining adequate physical distances. Data show variable rates of adherence to these recommendations across different countries. Some scholars argued that individuals might change their intention to adhere to public health prescriptions depending on socio-demographic characteristics: in particular, women with children have been found to be more compliant than males; work status has been reported to be associated with different levels of consciousness, as well as trust in government [2,3]; political judgment, due to polarization in the public debate, also exerts an influence on people’s compliance [4]; moreover, moral orientation and social values such as care values and perspective taking correlate with compliance correlate with adherence to public health prescriptions [5,6]. Finally, psychological factors also has been shown to be highly relevant [7]. For instance, the widespread of public fear for a health crisis could lead not only to high levels of psychological distress at the population level, but also to unhealthy behaviors, and this can undermine the effectiveness of communicational messages aimed at promoting public behavioral change [8]. As a consequence, excessive levels of emotional distress can lead the individuals towards maladaptive behaviors such as communication avoidance, risk negation and fear control strategies [9].

Thus, individuals’ emotional status has been recognized to be a crucial psychological factors in orienting people adherence to public health behavioral recommendation during emergency settings. Prior reviews highlight that individuals are at greater risk of experiencing feelings of anxiety and increased psychological distress during periods of quarantine or social restrictions due to pandemics [10]. Particularly, increased depressive and anxiety symptoms have been associated with poorer adherence to health-related recommendations, also during the COVID-19 pandemic [11,12].

Another well-known psychological determinant of citizens’ compliance towards public health recommendations is constituted by individuals’ self-efficacy in executing and following the precautionary measures. This is important, but already evidenced in social science literature showing that an individual’s perception of his/her competency to successfully perform the expected precautionary measures for COVID-19 affects his/her willingness and actual behavior to adhere to the measures themselves [13,14].

Thus, in a public emergency it is crucial to support citizens’ alliance with government and health authorities to promote public adherence to the containment measures. Public engagement during a health emergency is the base for enhancing public collaborations with governmental actions and reducing mass panic and maladaptive behavioral reactions. Thanks to public health engagement, governments can improve citizens’ resilience to the crisis and sustain more effective communication campaigns in order to make them understand the reasons behind governmental or healthcare choices [15].

Generally speaking, the concept of public engagement refers to people participation in political activities and in policy making [16,17]. In a wider sense, it refers to the general involvement of citizens in public affairs, and is the result of a trusted relationship between society and public authorities [18,19]. Public engagement is today considered a holistic approach to enhance citizens’ understanding of their crucial role in decisions and actions related to the management of the common goods [20,21]. In order to boost the alliance between public authorities and citizens in times of health crisis it is important not only to allow citizens to understand the public priorities and concerns, but also to monitor public fears and worries in order to avoid mass panic phenomena, public disagreement, and boycotting [22,23]. Promoting public engagement, health authorities can promote self-resilience in crisis response and build a trusted relationship which is functional to go beyond a simple information exchange [24–27]. Making citizens perceive themselves as active partners of the healthcare system during a public health crisis is a fundamental element to sustain the effectiveness and sustainability of preventative actions. In such moments, thus, monitoring the levels of public engagement of citizens becomes important. However, so far there is not agreement on the metrics and indexes to measures public engagement during a health crisis. A broad brunch of media studies, suggest to measure public engagement in terms of social media engagement index (i.e. in terms of popularity, reach and virality) of an health campaign message [28], simply calculated in terms of likes, followers, shares and repost on social media. Although these are technically considered objectives parameters of public engagement in media studies, and are often used in follow up studies [29,30], from a psychological perspective those could be considered only partial indexes of the public availability to trust a preventive message and to change behaviors accordingly.

In the area of chronic care, where the concept of patient engagement has been widely demonstrated to be a critical variable to improve adherence to treatment and to life style change [31–33], there is a broad agreement on the importance of measuring this psychological disposition in terms of the patients’ level of motivation, positive attitude, skills and emotional readiness to take action in their healthcare management [34].

Among the available patient engagement scientific measures, the Patient Health Engagement Scale (PHE-s^®^) is a validated instrument theoretically rooted in a robust psychological framework which explains the emotional process of engagement development [35]. This measure has been demonstrated to be reliable in chronic care to predict patients’ activation in self-management and adherence to treatment [31,36]. The assumption at the basis of this scale (and its related conceptual model) is that the level of engagement is function of the level of individual’s emotional adjustment to a critical health event, in the direction of mastering an increased sense of agency and control on one’s own cognitive and behavioral processes [37].

According to the Patient Health Engagement Model [35], the level of patients’ engagement might change in spite of critical events thanks to individual’s ability to give sense to this event or to cope with it. Previous studies have pointed out that the ability to be resilient in front of a public health crisis due–such as the COVID19 pandemic—and to maintain a sense of agency in spite to the emergency have constituted protective factors against maladaptive behaviors and non-adherence to preventive measures [35]. The Patient Health Engagement Scale–if adequately adapted to the crisis situation–may offer a useful instrument to measure the readiness of individuals to cope with it and to adhere to the prescribed behavioral change to contain the emergency; moreover it could provide authorities with useful information to target the population most at risk of non-adherence to the recommended behavioral measures.

**Fig 1:**
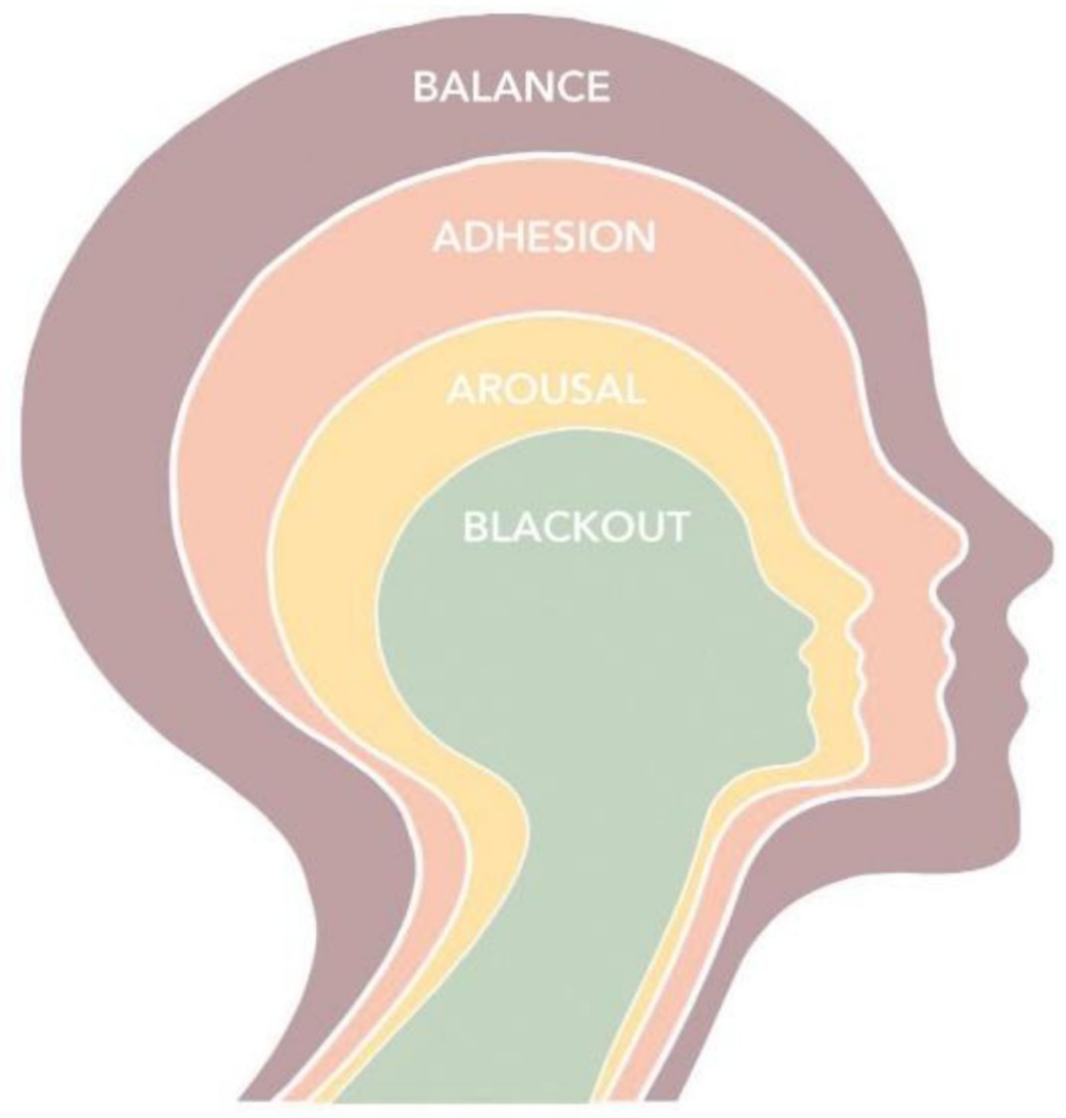
The public health engagement for emergency settings model.

More into details, the Patient Health Engagement model [35,38] is a psychological framework which explains how the level of patients health engagement depends on a process of continuous emotional and motivational reframing of how they perceive their own role in the management of a disease condition and its treatment (i.e. from passive receiver of prescriptions and services to active partner of the healthcare system). The model theorizes that an individual, in order to get fully engaged should be emotionally resilient and able to adjust to the health crisis and its specific requirements to prevent an illness situation. This model also features four psychological coping styles with health emergencies (such as the Covid-19 pandemic). The model features four positions: the first position (“Blackout”) is characterized by a state of psychological passivity and disengagement, typically occurring when people feel vulnerable and without control over the perceived risk, psychologically frozen and behaviorally paralyzed. The psychological position of “Arousal” follows. In this state people have acquired an initial awareness about their health risks but don’t master enough knowledge and competences to adequately face them. They do not accept the impact of preventive requirements on the modification of their daily habits and appear hyper-vigilant over their body signals/symptoms, hyperactive and confused when seeking information on the health emergency situation. Each unexpected news or change in the crisis situation causes psychological alert and overwhelming emotional responses, with disorganized actions and behaviors. When individuals succeed in the process of emotional regulation and coping with the stressful condition, they achieve a position of “Adhesion”. In this phase, patients have matured a good psychological adaptation to the critical situation and appear able to manage their psychological di-stress connected to health emergency. They appear more motivated to comply with medical and preventive prescriptions. In this phase, moreover, patients acquire further skills to effectively managing their risk condition. Finally, when people mature a complete awareness of the characteristics and consequences of the critical situation, and assume a better responsible position in their behaviors and risk management they reach the “Balance” phase, which features a better, positive and optimistic approach to the situation, with an increased ability to deal with the uncertainty of the moment and a strong motivation to psychologically achieve the sense of a “new normality” (Fig 1).

According to these premises, this study aims at proposing an adaptation and novel validation of the original PHE-s, intended for the use in health emergency and crisis settings (namely, the *Public Health Engagement Scale for Emergency Settings* (PHEs-E)).

More into details, the aims of this paper are to:

1. to investigate the psychometric properties of the revised version of the PHEs-E for emergency and crisis settings (reliability, validity);
2. describe how the construct of public health engagement as measured by the PHEs-E correlates with other psychological constructs; in particular with the presence of anxiety and depressive symptoms, and with the feeling of self-efficacy.
3. describe how the levels of public health engagement changed during the subsequent phases of the Covid-19 pandemic and across different sociodemographic groups.

## Methods

### Sample and procedure

This study is part of a broader project (“Italian Citizens’ Food Habits Monitoring from a Consumer Psychology Perspective) aimed at monitoring Italian citizens’ habits. Previous publications from this study [39,40] included an early developed scale for the measurement of public health engagement (*Public Health Engagement Scale for Emergency Settings* (PHEs-E)) and its first validation.

As already mentioned, the study consisted in a series of cross-sectional observations of 5 independent samples, each of about 1000 subjects, weighted to be representative of the Italian adult population (18–70 years old), recruited in 5 different moments of the COVID-19 pandemic in Italy.

In particular, data were collected:

1. Between February 28 and March 4, 2020: first COVID19 patient diagnosed in Italy and beginning of the epidemics in the country
2. Between May 12 and May 18, 2020: end of the first Italian nation-wide lockdown
3. Between September 20 and 25, 2020: after the summer, beginning of school activities and when the number of infections begun to rise again in Italy
4. Between November 27 and December 3, 2020: during the peak of the second wave of infections in Italy
5. Between March 12 and 17, 2021: during the third peak of infections in Italy

Participants were recruited by a professional panel provider (Norstat Italia), which employed a stratified sampling strategy controlled for gender, age, geographical area of residence, employment, and wage. After recruitment, participants were asked to provide an informed consent and to fill an online survey.

This study has been performed in accordance with the Declaration of Helsinki and has been approved by an independent ethics committee of Università Cattolica del Sacro Cuore in Milan (CERPS).

### Materials

The online survey included:

- The revised version of the Patient Health Engagement scale, featured in [39], for the purpose of revalidating it as the “*Public Health Engagement Scale for Emergency Settings* (PHEs-E)”. This scale features five items answered on a 7-points ordinal scale. The answering scale has, on the odd points, a short label describing a series of possible ways that a patient may feel like, while the even points are considered intermediate states. The labels on the right are associated with a higher engagement, while the labels on the left are associated with a lower engagement. The participants are requested to indicate the labels that better described how they feel “thinking about the emergency”, eventually using the intermediate points.
- The Self-rating Depression Scale (SDS) (only from wave 3), a measure for depression consisting in 20 items answered with a Likert type scale ranging from 1 to 4. Higher scores indicate a greater presence of depressive symptoms [41];
- The Self-rating Anxiety Scale (SAS) (only from wave 3), a measure for anxiety disorders consisting in 20 items answered with a Likert type scale ranging from 1 to 4. Higher scores indicate a greater presence of anxiety symptoms [42];
- The Generalized Self-Efficacy scale (only from wave 2) is a 10-items measure designed to assess optimistic self-beliefs on the ability to cope with difficult demands in life [43];
- A series of socio-demographic questions to assess gender, age, level of education, geographical area of residence, wage, employment and whether they suffered or not from a chronic condition.

### Statistical analyses

Descriptive statistics were carried out to assess the distribution of socio-demographic variables in the sample and the distribution of answers in the 5 items.

The five ordinal items of the PHEs-E were then recoded from a 7-points scale to a 4-points scale. In particular, for the purpose of these analyses and of the scoring, intermediate points (which don’t have a proper label) are considered as if the participant answered the previous point (i.e., 1&2 are recoded as 1, 3&4 are recoded as 2, 5&6 are recoded as 3 and 7 is recoded as 4).

Then, a Confirmatory Factor Analysis (CFA) was performed to assess the scale monofactoriality. Goodness of fit indices were evaluated: in particular, as suggested by Hu & Bentler [44], we considered a good model fit a root-mean-square error of approximation (RMSEA) and its 90% confidence interval below <0.06; a standardized root mean square residual (SRMR) <0.08; and a Comparative Fit Index >0.95. Additionally, as suggested by Schumacker & Lomax [45] we considered an index of good fit a χ^2^/degrees of freedom (df) ratio close to or below 5. Maximum Likelihood (ML) estimator with bootstrapped error estimates (5.000 samples) was used.

Finally, to evaluate the psychometric properties of the HE scale, a Partial Credit Rasch Model (PCM) was performed to further check uni-dimensionality and the fit of each ordinal item at the construct of interest [46]. In particular, PCM was chosen because the items (once recoded) have four response options and showed different patterns of usage (namely, the distance between each step is different between the different items) [47,48]. The parameters of the Rasch model were estimated by the ML method [49]. Then, the Person Separation Index (PSI) was calculated to evaluate the PCM reliability. Values of PSI superior to 0.8 are generally considered acceptable [50,51], and indicate a good reproducibility of the measured location of the persons. Moreover, to check whether the items fitted the expected model, Infit and Outfit mean square (MNSQ) statistics were computed. If the data fit the PCM, the fit statistics are expected to lie in a range between 0.6 and 1.4 [52]. Analyses of difficulty and step parameters were conducted to guarantee a sufficient ranking of the different categories of response and to respect the monotonic order. Finally, Empirical Ordinal Alpha [53] was also calculated, due to the ordinal nature of the items, as a measure of internal consistency. An index superior to 0.7 interpreted as acceptable, while an index around 0.9 is considered excellent [54].

In order to assess the variation of PHEs-E across waves and across different sociodemographic groups a General Linear Model (GLM) was performed. The dependent variable of the GLM was the Public Health Engagement score; the main effects wave (from 1 to 5), gender (male/female), having a chronic disease (yes/no), wage (below or above median), and education (middle school, high school, university) were calculated, and post-hoc analyses were run when appropriate. Moreover, two-way interaction terms between “wave” and each sociodemographic variable were added to the model. We refrained from computing interactions between sociodemographic variables (e.g. gender*education), as well interactions between more factors, as this was beyond the purpose of this analysis. Participants who didn’t provide information regarding their wage (14.9% of the whole sample) were removed from this analysis, as their data were incomplete.

Furthermore, the model was corrected by age, as many factors are likely to be also associated with a difference in age.

Finally, Pearson’s linear correlations between the level of PHEs-E and the scores of the SAS, SDS and Self-efficacy scales were computed both in the whole sample and in each wave.

All statistical analyses were conducted using IBM SPSS v27 and Amos v21, with the only exception of Ordinal Alpha, which was calculated using R.

## Results

### Sample characteristics

The whole sample amounts to 4981 Italian citizens (50.8% female) aged between 18 and 70 years old (mean 45 with a standard deviation of 14). Table 1 shows the socio-demographic characteristics of the 5 independent samples.

**Table 1:** Samples characteristics.

	%				
	Wave 1	Wave 2	Wave 3	Wave 4	Wave 5
Gender					
Male	48.9	49.2	48.6	49.2	49.6
Female	51.1	50.8	51.4	50.8	50.4
Employment					
Entrepreneur/freelancer	7.0	7.6	8.3	9.4	9.4
Manager/official	2.0	1.4	2.4	1.6	2.1
Employee/military/teacher	19.4	21.7	22.9	23.6	24.3
Worker/shop assistant/apprentice	22.4	22.6	22.4	21.4	23.0
Householder	14.9	14.1	14.2	15.2	15.2
Student	6.8	6.8	6.9	6.9	7.0
Retired	8.5	8.5	8.9	9.2	9.6
Unoccupied	17.0	17.3	14.1	12.8	9.6
Other	2.0	0.0	0.0	0.0	0.0
Education					
Middle school or lower	15.3	13.6	13.2	13.6	15.1
High school	61.6	61.0	60.8	58.8	59.5
University degree or higher	23.1	25.4	26.0	27.6	25.4
Wage					
Below median (1800€/month)	48.8	52.1	46.7	43.2	45.5
Above median (1800€/month)	36.7	32.9	37.8	41.1	41.7
Missing (not answered)	14.6	15.0	15.6	15.7	12.8
Has a chronic disease					
No	81.5	82.0	84.5	83.7	83.9
Yes	18.5	18.0	15.5	16.3	16.1
Geographical Area					
North-west	26.1	26.3	26.4	27.4	27.6
North-east	18.4	18.4	18.5	17.1	17.8
Center	20.0	20.1	19.8	18.6	19.7
South	35.4	35.2	35.2	36.9	34.9

### PHEs-E validation

A confirmatory factorial analysis was run to assess the PHEs-E assumed mono-factoriality. Table 2 shows the standardized regression weights between the latent construct and the observed items. All the observed items’ variabilities seem to be well explained by the latent factor, with standardized estimates ranging between 0.668 and 0.776.

**Table 2:** Standardized regression weights in the first CFA.

Items	Standardized Estimate	p-values
PHEs-E_1	0.668	<.001
PHEs-E_2	0.769	<.001
PHEs-E_3	0.748	<.001
PHEs-E_4	0.776	<.001
PHEs-E_5	0.692	<.001

Generally speaking, the model fit was acceptable, although not completely satisfactory: χ^2^_(df = 5)_ = 485.449, p <.001; χ^2^/df = 97.09; CFI = 0.953; RMSEA = 0.139 (90% C.I.: 0.129–0.149); SRMR = 0.0391. In particular, the significant χ^2^, the high ratio between χ^2^ value and degrees of freedom, and the high RMSEA index suggested a possibly poor fit. An inspection of the modification indexes revealed a strong correlation between the errors of Item 1 and Item 2 (Modification Index = 374.640). Instead of correlating the errors to increase model fit, we decided to remove the Item with the lowest factor loading (Item 1), as the high modification index suggests a redundancy between the two items, making one of them unnecessary.

A second CFA was thus run with Item 1 removed, Table 3 shows standardized regression weights between the latent construct and the observed items for this second model. The standardized factor loadings in this second model ranged between 0.705 and 0.799.

**Table 3:** Standardized regression weights in the second CFA.

Items	Standardized Estimate	p-values
PHEs-E_2	0.712	<.001
PHEs-E_3	0.774	<.001
PHEs-E_4	0.799	<.001
PHEs-E_5	0.705	<.001

In this case, after removing the redundant item, the model fit was fully satisfactory according to Hu and Bentler’s suggestions [44]: χ^2^_(df = 2)_ = 11.422, p = .003; χ^2^/df = 5.71; CFI = 0.999; RMSEA = 0.031 (90% C.I.: 0.015–0.049); SRMR = 0.007.

Table 4 shows the results of the PCRM to test the psychometric properties of the PHEs-E scale.

**Table 4:** Results of the partial credit rasch model.

	Location	Step 1	Step 2	Step 3	Outfit	Infit
PHEs-E_2	1.844	-2.469	1.502	6.499	0.787	0.817
PHEs-E_3	0.808	-2.672	1.188	3.907	0.688	0.693
PHEs-E_4	1.500	-1.922	1.467	4.954	0.653	0.660
PHEs-E_5	0.891	-2.471	-0.221	5.365	0.698	0.742

The item statistics ranged from .660 to 0.817 for the infit MSQ and from .653 to 0.787 for the outfit MSQ. These values indicate an acceptable fit of the Rasch Model. The distances between subsequent thresholds showed acceptable distinction between the response options and measurement model fit. The PSI for revised HE scale was equal to 0.788, thus close to the acceptability range. Rasch Model confirmed the unidimensionality of the revised PHEs-E and the fit of each item of the scale to the data.

Finally, the PHEs-E scale showed a good internal consistency, as empirical ordinal alpha was equal to 0.77.

### Descriptive statistics, scoring and cut-offs

After normalizing the Rasch scores to fit into a 0–100 scale, the scores show a rather normal distribution with mean 52.95, standard deviation of 19.72, skewness of -0.002 and kurtosis of -0.022.

Four groups were then identified, namely below -1 std. deviation, between -1 std. deviation and the mean, between the mean and +1 std. deviation, and above +1 standard deviation. Table 5 shows the percentage of participants in each group.

**Table 5:** Percentage of participants in each PHEs_E group.

PHEs_E group	% in the sample
1-Blackout	18.5
2-Arousal	23.3
3-Adhesion	45.7
4-Balance	12.5

### PHEs-E changes over time

The results from the GLM showed a significant main effect of wave (F_(4, 4536)_ = 46.721; p <.001; η_p_^2^ = 0.040). In particular, Bonferroni post-hoc tests revealed that public health engagement was significantly higher with p <.001 in the first wave then in the other waves. PHEs-E in wave 2 resulted equal that in wave 3, and in both it resulted higher than in wave 4 and 5. PHEs-E in wave 4 was equal to PHEs-E in wave 5, and in both wave 4 and 5 it resulted lower than in the other waves. PHEs-E marginal means corrected for age were 61.00, 52.74, 53.01, 47.71 and 44.85 respectively for waves from 1 to 5. Additionally, results from the GLM showed a significant main effect of gender (F_(1, 4536)_ = 57.170; p <.001; η_p_^2^ = 0.012). In particular, male participants resulted having a higher PHEs-E (M = 54.01) than female participants (M = 49.75) with p <.001. Moreover, results show a significant main effect for having a chronic disease as well (F_(1, 4536)_ = 11.668; p = .001; η_p_^2^ = 0.003). In particular, participants with a diagnosed chronic disease had a lower level of PHEs-E (M = 53.19) when compared with participants not diagnosed with a chronic disease (M = 50.57). No other main effect resulted significant.

**Fig 2:**
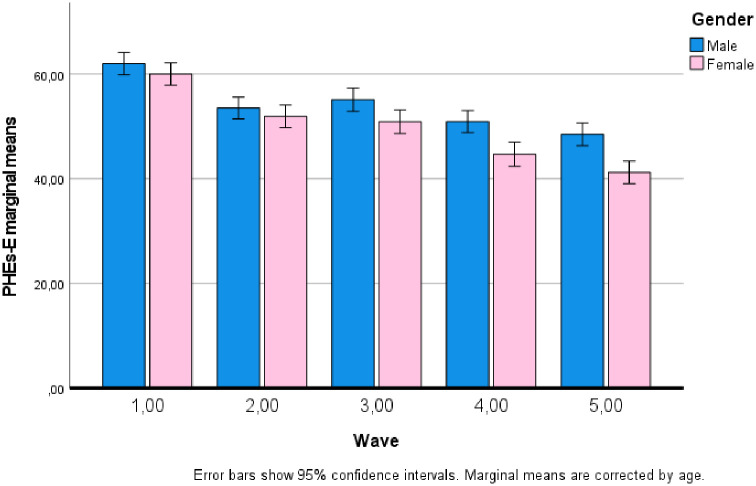
Interaction between gender and wave on PHEs-E marginal means.

Finally, a two-way interaction effect between wave and gender resulted statistically significant (F_(4, 4536)_ = 4.013; p = .003; η_p_^2^ = 0.004). In particular, post-hoc tests show that while in wave 1 and wave 2 there was no statistically significant difference between males and females, in waves 3, 4, and 5 males show a significantly (with p <.001) higher PHEs-E score than females (differences in marginal means 4.23, 6.24, and 7.25 respectively for wave 3, 4, and 5). Fig 2 shows the interaction of gender by wave. No other significant interaction was found.

### Correlations with other constructs

Results show that PHEs-E has a strong negative correlation with both anxiety (r = -0.506, p <.001) and depression (r = -0.509, p <.001). Instead, it has a moderate, positive correlation with self-efficacy (r = 0.293, p <.001). Those correlations seem to be consistent across the 5 waves of data collection, as Table 6 shows.

**Table 6:** Correlations with PHE across waves and in the whole sample.

Waves of data collection	Constructs and correlation indexes with PHEs-E		
	GSE	SAS	SDS
Wave 2	0.294 *	n/a	n/a
Wave 3	0.349 *	-0.494 *	-0.474 *
Wave 4	0.244 *	-0.531 *	-0.532 *
Wave 5	0.299 *	-0.475 *	-0.507 *
Whole sample	0.293 *	-0.506 *	-0.509 *

* significative at p <.001 level.

## Discussion

During a health emergency or a crisis situation, being able to guarantee the collaboration of citizens to protective measures imposed by health authorities is a crucial requirement. However, fostering citizens’ awareness about the health risks and their collaboration with public health authorities is not a simple task. The extent of communities’ active collaboration in managing health risks and their level of compliance to proposed public health measures require continuous monitoring in order to address effective campaigns of communication and education. During the COVID19 pandemic this aspect became particularly evident: engaging citizens in changing their behaviors and in adhering to the contagion containment measures has been the unique weapon against the pandemic for many months [1,55].

However, the level of citizens’ motivation to partnering with the healthcare authorities during a pandemic or a health emergency cannot be given for granted. Psychological factors frame it and may hinder or foster individuals’ ability to cope with the emergency and to adhere to public health requirements.

A part from the level of health literacy [56], the kind of individuals’ emotional reaction to the health emergency has been demonstrated to be an important predictor of individuals’ engagement in the crisis management. However so far, not dedicated measurement exists able to assess the psychological extent to which individuals are ready to get engaged in the management of a health emergency [57,58].

In order to offer a contribute towards the measurement of the psychological reactions to a health crisis which may hinder or sustain individuals’ engagement in public health measures, in this study we prosed an adaptation and validation of the Patient Health Engagement Scale in Emergency Settings (PHEs-E).

Results from the confirmatory analysis and the partial credit Rasch model both support the mono-dimensionality of the 4-items scale. Empirical ordinal alpha shows a good internal consistency.

Furthermore, the scale showed a good variability in time, by demonstrating its ability to track emergency differences in the psychological reactions of individuals to the COVID19 epidemiological situations and related measures of containment of the virus spread. This element is an important asset in order to equip public health authorities with indexes able to monitor emotional status and behavioral change attitudes of a population in reaction to public health communication campaign. In the specific case of the different phases of the COVID19 emergency in Italy, finds coming from our monitoring of the PHEs-E underline a worrying phenomenon: the average levels of the PHEs-E in the Italian population decreased over time, thus suggesting an ineffectively of the strategies enabled by Italian public health authority in sustaining people motivation to adhere to the containment measures. This data is also an interesting indicator of the impact of lockdown measures and of the prolonged state of emergency on the psychological status of the population, by causing emotional frustration, sense of exhaustion and increased sense of fatalism [59].

Consistently with this interpretation, PHEs-E levels appears inversely correlated with the levels of individuals’ anxiety and depression: this confirm the ability of the PHEs-E in depicting the emotional status of the population facing a health emergency and being an indicator of individuals psychological copying ability. Previous literature, indeed, has demonstrated how the extent of psychological resilience [60,61] such as the ability to emotionally cope with an expecting and frightening health event is crucial in order to sustain individuals’ compliance to public health measures during a pandemic [62].

The slight correlation between PHEs-E and gender is interesting and worthy further explorations: previous studies in the area of chronic care management and in the area of primary prevention report contradictory results related to the role of gender in determining individuals’ level of engagement in adherence to behavioral change requirements [63,64]. In the specific case of the COVID19 pandemic, due to the overwhelming emotional burden that this emergency and the related containment measures implied, individuals’ ability to keep under control one own psychological reaction resulted a crucial asset for fostering behavioral engagement in health prevention. Previous literature suggests that emotional balance and control in a crisis situation is often related to gender and that, although with some cultural variation, men tend to be more resilient and able to control their emotional reactions [65].

Finally, the positive correlation between PHEs-E and the levels of individuals’ Self-Efficacy confirms the applicability of this index to measure the extent to which individuals perceive themselves as crucial partners of the healthcare authorities in the management on a health crisis. Self-Efficacy, indeed, has been largely demonstrated to be a predictor of individuals’ motivation to enact health behaviors change and of adherence to preventive measures [66,67]: the extent to which people perceive themselves as able to perform a preventive behavior influence their inclination to adopt it. Even in the case of a public health emergency, assessing how much citizens believe themselves able to cope with it and to adopt the preventive measures suggested by the health authorities is important in order not only to predict their levels of engagement but also to orient personalized public health communication campaigns to sustain their perceived self-efficacy and motivation in behavioral change.

### Limitations and future studies

This study has a few limitations. First, the employed methodology is not properly longitudinal, as single subjects were not tracked over time individually, and the 5 samples from the different waves were thus independent. For this reason, inferences regarding changes of the observed construct (Public Health Engagement) over time should be regarded with some caution, as this would require a single sample being tracked over time. Nevertheless, the 5 samples were designed to be comparable and equally representative of the Italian population, which supports the reliability and the relevance of our data and analyses.

Moreover, not all the variables were measured in all waves: the construct of anxiety and depression, for instance, was included along the course of the study on the basis of the clear evidence of psychological sufferance in the Italian population due to COVID19 and containment measures. This change in the structure of the questionnaire does not allow a full comparability of datasets across the 5 periods of data collection and reduced the amount of complete data.

Finally, this study provides no evidences of the association between the proposed construct of Public Health Engagement and actual preventive behaviors. Future studies should aim at addressing this limitation, as the current lack of evidences only permits to associate the construct with attitudes and intentions, and not with actual behaviors. Thus, it is still to be explored the gap between Public Health Engagement, intention to adhere to preventive behaviors, and the actual behaviors.

### Practical implications

This study’s findings indicate that individuals’ characteristics may differentiate citizens’ motivation to engage in public health behavioral recommendation to prevent the COVID-19 contagion. However the scale could be useful to perform a psychological monitoring of psychological readiness to engage in public health strategies to face critical events and settings. In particular, the level of public health engagement appears to be a crucial construct to understand changes in individuals coping with health emergencies. Accordingly, the provision of resources should be prioritized for citizens, to address psychological concerns during and after the pandemic. Our findings suggest that being engaged in adopting new preventive health behaviors may modulate the risks of COVID-related fears and worries on depressive and anxiety symptoms. While we showed this with our sample, such behaviors should be considered in relation to understanding mental health across a broader population. Preventive health behaviors have been broadly advocated throughout the COVID-19 pandemic, and these behaviors may be a useful means in helping individuals cope with daily hassles beyond addressing the contagion. These results highlight the importance of these psychosocial factors in shaping community responses to a pandemic. The findings also offer insights into potential targets for public health communication aiming at promoting public health engagement in preventive measures.

Moreover, public health communication strategies should maximize their impact if personalized according to the public health engagement levels of citizens. For instance, in order to improve the levels of engagement of citizens in a “psychological blackout,” it appears fundamental to opt for a tone of voice which may reassure the target and sustain their emotional elaboration of the critical event in order to motivate them to be calm and adherent. reassuring messages aimed at sustaining the emotional elaboration of the emergency and related worries would be particularly needed. To enhance the motivation to stay engaged, citizens in a situation of “psychological adherence” would need messages which boost their sense of hope and self-efficacy: testimonials of peers, positive stories of other persons who succeeded in adhering to the prescribed containment measures sharing tips and pragmatic solutions may be useful. Finally, people in the position of “Balance”, deserve messages which help in maintaining new sense of normality despite crisis; moreover they may be engaged as positive advocates of a correct adherence to public health measures and can be involved as testimonial or allied of the health authorities to sensitize their peers about a correct approach to the emergency.

## Supporting information

S1 File Study data. Study data in .sav format.
(SAV)

## References

[1] Van Bavel JJ, Baicker K, Boggio PS, Capraro V, Cichocka A, Cikara M, et al. Using social and behavioural science to support COVID-19 pandemic response. Nat Hum Behav. 2020;4: 460–471. doi: 10.1038/s41562-020-0884-z 32355299

[2] Uddin S, Imam T, Khushi M, Khan A, Moni MA. How did socio-demographic status and personal attributes influence compliance to COVID-19 preventive behaviours during the early outbreak in Japan? Lessons for pandemic management. Pers Individ Dif. 2021;175: 110692. doi: 10.1016/j.paid.2021.110692 33526954 PMC7839830

[3] Kabamba Nzaji M, Ngoie Mwamba G, Mbidi Miema J, Kilolo Ngoy Umba E, Kangulu IB, Banza Ndala DB, et al. Predictors of Non-Adherence to Public Health Instructions During the COVID-19 Pandemic in the Democratic Republic of the Congo. J Multidiscip Healthc. 2020;Volume 13: 1215–1221. doi: 10.2147/JMDH.S274944 33116566 PMC7585776

[4] Hart PS, Chinn S, Soroka S. Politicization and Polarization in COVID-19 News Coverage. Sci Commun. 2020;42: 679–697. doi: 10.1177/1075547020950735 PMC7447862 38602988

[5] Amin AB, Bednarczyk RA, Ray CE, Melchiori KJ, Graham J, Huntsinger JR, et al. Association of moral values with vaccine hesitancy. Nat Hum Behav. 2017;1: 873–880. doi: 10.1038/s41562-017-0256-5 31024188

[6] Díaz R, Cova F. Reactance, morality, and disgust: the relationship between affective dispositions and compliance with official health recommendations during the COVID-19 pandemic. Cogn Emot. 2021; 1–17. doi: 10.1080/02699931.2021.1941783 34132171

[7] Hagger MS, Smith SR, Keech JJ, Moyers SA, Hamilton K. Predicting Social Distancing Intention and Behavior During the COVID-19 Pandemic: An Integrated Social Cognition Model. Ann Behav Med. 2020;54: 713–727. doi: 10.1093/abm/kaaa073 32914831 PMC7543267

[8] Anaki D, Sergay J. Predicting health behavior in response to the coronavirus disease (COVID-19): Worldwide survey results from early March 2020. PLoS One. 2021;16: e0244534. doi: 10.1371/journal.pone.0244534 33411827 PMC7790278

[9] Noone C, Warner NZ, Byrne M, Durand H, Lavoie KL, McGuire BE, et al. A scoping review of research on the determinants of adherence to social distancing measures during the COVID-19 pandemic. Health Psychol Rev. 2021;15: 350–370. doi: 10.1080/17437199.2021.1934062 34027798

[10] Brooks SK, Webster RK, Smith LE, Woodland L, Wessely S, Greenberg N, et al. The psychological impact of quarantine and how to reduce it: rapid review of the evidence. Lancet. 2020;395: 912–920. doi: 10.1016/S0140-6736(20)30460-8 32112714 PMC7158942

[11] Segrin C, Passalacqua SA. Functions of Loneliness, Social Support, Health Behaviors, and Stress in Association With Poor Health. Health Commun. 2010;25: 312–322. doi: 10.1080/10410231003773334 20512713

[12] Stickley A, Matsubayashi T, Sueki H, Ueda M. COVID-19 preventive behaviours among people with anxiety and depressive symptoms: findings from Japan. Public Health. 2020;189: 91–93. doi: 10.1016/j.puhe.2020.09.017 33189941 PMC7547627

[13] Chong YY, Chien WT, Cheng HY, Chow KM, Kassianos AP, Karekla M, et al. The Role of Illness Perceptions, Coping, and Self-Efficacy on Adherence to Precautionary Measures for COVID-19. Int J Environ Res Public Health. 2020;17: 6540. doi: 10.3390/ijerph17186540 32911779 PMC7558870

[14] Sheeran P, Maki A, Montanaro E, Avishai-Yitshak A, Bryan A, Klein WMP, et al. The impact of changing attitudes, norms, and self-efficacy on health-related intentions and behavior: A meta-analysis. Heal Psychol. 2016;35: 1178–1188. doi: 10.1037/hea0000387 27280365

[15] Gálvez-Rodríguez M del M, Haro-de-Rosario A, García-Tabuyo M, Caba-Pérez C. Building online citizen engagement for enhancing emergency management in local European government. Online Inf Rev. 2019;43: 219–238. doi: 10.1108/OIR-09-2016-0286

[16] Gaventa J, Barrett G. Mapping the Outcomes of Citizen Engagement. World Dev. 2012;40: 2399–2410. doi: 10.1016/j.worlddev.2012.05.014

[17] Siebers V, Gradus R, Grotens R. Citizen engagement and trust: A study among citizen panel members in three Dutch municipalities. Soc Sci J. 2019;56: 545–554. doi: 10.1016/j.soscij.2018.09.010

[18] Agostino D, Arnaboldi M. A Measurement Framework for Assessing the Contribution of Social Media to Public Engagement: An empirical analysis on Facebook. Public Manag Rev. 2016;18: 1289–1307. doi: 10.1080/14719037.2015.1100320

[19] Taylor M, Kent ML. Dialogic Engagement: Clarifying Foundational Concepts. J Public Relations Res. 2014;26: 384–398. doi: 10.1080/1062726X.2014.956106

[20] Stilgoe J, Lock SJ, Wilsdon J. Why should we promote public engagement with science? Public Underst Sci. 2014;23: 4–15. doi: 10.1177/0963662513518154 24434705 PMC5753839

[21] Leshner AI. Public engagement with science. (Editorial). Science. American Association for the Advancement of Science; 2003. p. 977. https://go.gale.com/ps/i.do?p=AONE&sw=w&issn=00368075&v=2.1&it=r&id=GALE%7CA98135987&sid=googleScholar&linkaccess=fulltext. 10.1126/science.299.5609.977 12586907

[22] Chatfield AT, Scholl HJ (Jochen), Brajawidagda U. Tsunami early warnings via Twitter in government: Net-savvy citizens’ co-production of time-critical public information services. Gov Inf Q. 2013;30: 377–386. doi: 10.1016/j.giq.2013.05.021

[23] Graham MW, Avery EJ, Park S. The role of social media in local government crisis communications. Public Relat Rev. 2015;41: 386–394. doi: 10.1016/j.pubrev.2015.02.001

[24] Palamenghi L, Barello S, Boccia S, Graffigna G. Mistrust in biomedical research and vaccine hesitancy: the forefront challenge in the battle against COVID-19 in Italy. Eur J Epidemiol. 2020;35: 785–788. doi: 10.1007/s10654-020-00675-8 32808095 PMC7431109

[25] Wang H, Cleary PD, Little J, Auffray C. Communicating in a public health crisis. Lancet Digit Heal. 2020;2: e503. doi: 10.1016/S2589-7500(20)30197-7 32838253 PMC7417177

[26] Carrera J, Key K, Bailey S, Hamm J, Cuthbertson C, Lewis E, et al. Community Science as a Pathway for Resilience in Response to a Public Health Crisis in Flint, Michigan. Soc Sci. 2019;8: 94. doi: 10.3390/socsci8030094

[27] Wulff K, Donato D, Lurie N. What is health resilience and how can we build it? Annu Rev Public Health. 2015;36: 361–374. doi: 10.1146/annurev-publhealth-031914-122829 25581148

[28] Bonsón E, Bednárová M. The use of YouTube in western European municipalities. Gov Inf Q. 2018;35: 223–232. doi: 10.1016/j.giq.2018.04.001

[29] Chen Q, Min C, Zhang W, Wang G, Ma X, Evans R. Unpacking the black box: How to promote citizen engagement through government social media during the COVID-19 crisis. Comput Human Behav. 2020;110: 106380. doi: 10.1016/j.chb.2020.106380 32292239 PMC7151317

[30] Denktaş-Şakar G, Sürücü E. Stakeholder engagement via social media: an analysis of third-party logistics companies. Serv Ind J. 2020;40: 866–889. doi: 10.1080/02642069.2018.1561874

[31] Graffigna G, Barello S, Bonanomi A. The role of Patient Health Engagement model (PHE-model) in affecting patient activation and medication adherence: A structural equation model. PLoS One. 2017;12. doi: 10.1371/journal.pone.0179865 28654686 PMC5487073

[32] Zullig LL, Bosworth H. Engaging Patients to Optimize Medication Adherence. NEJM Catal. 2017;14: 1–14. Available: https://catalyst.nejm.org/optimize-patients-medication-adherence/.

[33] Bombard Y, Baker GR, Orlando E, Fancott C, Bhatia P, Casalino S, et al. Engaging patients to improve quality of care: A systematic review. Implement Sci. 2018;13. doi: 10.1186/s13012-018-0784-z 30045735 PMC6060529

[34] James J. Patient Engagement. Heal Policy Br. 2013 Feb. doi: 10.1377/hpb20130214.898775

[35] Graffigna G, Barello S. Spotlight on the patient health engagement model (PHE model): A psychosocial theory to understand people’s meaningful engagement in their own health care. Patient Preference and Adherence. 2018. pp. 1261–1271. doi: 10.2147/PPA.S145646 30050288 PMC6056150

[36] Graffigna G, Barello S, Bonanomi A, Riva G. Factors affecting patients’ online health information-seeking behaviours: The role of the Patient Health Engagement (PHE) Model. Patient Educ Couns. 2017. doi: 10.1016/j.pec.2017.05.033 28583722

[37] Graffigna G, Barello S, Bonanomi A, Lozza E. Measuring patient engagement: Development and psychometric properties of the patient health engagement (PHE) scale. Front Psychol. 2015;6: 1–10. doi: 10.3389/fpsyg.2015.00001 25870566 PMC4376060

[38] Barello S, Graffigna G. Patient engagement in healthcare: Pathways for effective medical decision making. Neuropsychol Trends. 2015;17: 53–65. doi: 10.7358/neur-2015-017-bare

[39] Graffigna G, Barello S, Savarese M, Palamenghi L, Castellini G, Bonanomi A, et al. Measuring Italian citizens’ engagement in the first wave of the COVID-19 pandemic containment measures: A cross-sectional study. Tu W-J, editor. PLoS One. 2020;15: e0238613. doi: 10.1371/journal.pone.0238613 32915822 PMC7485890

[40] Graffigna G, Palamenghi L, Savarese M, Castellini G, Barello S. Effects of the COVID‐19 Emergency and National Lockdown on Italian Citizens’ Economic Concerns, Government Trust, and Health Engagement: Evidence From a Two‐Wave Panel Study. Milbank Q. 2021;0: 1468–0009.12506. doi: 10.1111/1468-0009.12506 33822424 PMC8241271

[41] Zung WWK. Zung Self-Rating Depression Scale and Depression Status Inventory. Assess Depress. 1986; 221–231. doi: 10.1007/978-3-642-70486-4_21

[42] Zung WWK. A Rating Instrument For Anxiety Disorders. Psychosomatics. 1971;12: 371–379. doi: 10.1016/S0033-3182(71)71479-0 5172928

[43] Schwarzer R, Jerusalem M. Generalized Self-Efficacy scale. Measures in health psychology: A user’s portfolio Causal and control beliefs. Windsor, UK: NFER-NELSON; 1995. pp. 35–37.

[44] Hu L, Bentler PM. Cutoff criteria for fit indexes in covariance structure analysis: Conventional criteria versus new alternatives. Struct Equ Model A Multidiscip J. 1999;6: 1–55. doi: 10.1080/10705519909540118

[45] Schumacker RE, Lomax RG. A Beginner’s Guide to Structural Equation Modeling—second edition. Mahwan: LAWRENCE ERLBAUM ASSOCIATES; 2004.

[46] Andrich D. A structure of index and causal variables. Rasch Measurement Transactions. 2014. pp. 1475–1477.

[47] Masters GN. A rasch model for partial credit scoring. Psychometrika. 1982;47: 149–174. doi: 10.1007/BF02296272

[48] Bonanomi A, Osmetti SA. THE RASCH MODEL FOR VICTIMIZATION ANALYSIS: A PROPOSAL OF AN INSECURITY PERCEPTION INDEX. Electron J Appl Stat Anal Decis Support Syst Serv Eval. 2012;3: 75–85. doi: 10.1285/i2037-3627v3n1p75

[49] Andrich D, Sheridan B, Lyne A, Luo G. RUMM: A windows-based item analysis program employing Rasch unidimensional measurement models. Perth, Australia: Murdoch University. Perth, WA; 2000.

[50] Wright BD, Masters GN. Rating Scale Analysis. Advances in Measurement in Educational Research and Assessment. Chicago, IL: MESA Press; 1982.

[51] Prieto L, Alonso J, Lamarca R. Classical Test Theory versus Rasch analysis for quality of life questionnaire reduction. Health Qual Life Outcomes. 2003;1: 27. doi: 10.1186/1477-7525-1-27 12952544 PMC194220

[52] Wright BD, Linacre JM, Gustafson JE, Martin-Lof P. Reasonable mean-square fit values. Rasch Meas Trans. 1994;8: 370.

[53] Bonanomi A, Cantaluppi G, Nai Ruscone M, Osmetti SA. A new estimator of Zumbo’s Ordinal Alpha: a copula approach. Qual Quant. 2015;49: 941–953. doi: 10.1007/s11135-014-0114-8

[54] Gliem JA, Gliem RR. Calculating, Interpreting, and Reporting Cronbach’s Alpha Reliability Coefficient for Likert-Type Scales. Midwest Research-to-Practice Conference in Adult, Continuing, and Community Education. Columbus, OH, USA: The Ohio State University press; 2003. doi: 10.1007/s00424-003-1026-y

[55] Betsch C, Böhm R, Chapman GB. Using Behavioral Insights to Increase Vaccination Policy Effectiveness. Policy Insights from Behav Brain Sci. 2015;2: 61–73. doi: 10.1177/2372732215600716

[56] Paakkari L, Okan O. COVID-19: health literacy is an underestimated problem. Lancet Public Heal. 2020;5: e249–e250. doi: 10.1016/S2468-2667(20)30086-4 32302535 PMC7156243

[57] Pakpour AH, Griffith MD. The fear of COVID-19 and its role in preventive behaviors. J Concurr Disord. 2020;2: 58–63. Available: http://irep.ntu.ac.uk/id/eprint/39561/ 33195740

[58] Latkin CA, Dayton L, Moran M, Strickland JC, Collins K. Behavioral and psychosocial factors associated with COVID-19 skepticism in the United States. Curr Psychol. 2021 [cited 3 Sep 2021]. doi: 10.1007/s12144-020-01211-3 33424206 PMC7786141

[59] Prati G, Mancini AD. The psychological impact of COVID-19 pandemic lockdowns: A review and meta-analysis of longitudinal studies and natural experiments. Psychological Medicine. Psychol Med; 2021. pp. 201–211. doi: 10.1017/S0033291721000015 33436130 PMC7844215

[60] Benham TL, Hart A, Bortolin M, Court M, Groves J, Kraus A, et al. Preparing for the Second Surge: Preventing Posttraumatic Stress Disorder and Building Resilience for Health Care Workers in the Face of COVID-19. Disaster Med Public Health Prep. 2020; 1–4. doi: 10.1017/dmp.2020.371 33046178 PMC7684017

[61] Fransen J, Peralta DO, Vanelli F, Edelenbos J, Olvera BC. The emergence of Urban Community Resilience Initiatives During the COVID-19 Pandemic: An International Exploratory Study. Eur J Dev Res. 2021; 1–23. doi: 10.1057/s41287-020-00348-y 33456209 PMC7802407

[62] Goodwin R, Wiwattanapantuwong J, Tuicomepee A, Suttiwan P, Watakakosol R. Anxiety and public responses to covid-19: Early data from Thailand. J Psychiatr Res. 2020;129: 118–121. doi: 10.1016/j.jpsychires.2020.06.026 32912591 PMC7326441

[63] Etingen B, Miskevics S, Malhiot A, LaVela SL. Patient Engagement in VA Health Care: Does Gender Play a Role? Def Peace Econ. 2020;31: 24–33. doi: 10.1080/10242694.2018.1465676

[64] Galdas PM, Harrison AS, Doherty P. Gender differences in the factors predicting initial engagement at cardiac rehabilitation. Open Hear. 2018;5: e000764. doi: 10.1136/openhrt-2017-000764 29632680 PMC5888444

[65] Fujita F, Diener E, Sandvik E. Gender differences in negative affect and well-being: The case for emotional intensity. J Pers Soc Psychol. 1991;61: 427–434. doi: 10.1037//0022-3514.61.3.427 1941513

[66] Strecher VJ, McEvoy DeVellis B, Becker MH, Rosenstock IM. The Role of Self-Efficacy in Achieving Health Behavior Change. Heal Educ Behav. 1986;13: 73–92. doi: 10.1177/109019818601300108 3957687

[67] Schwarzer R, Renner B. Social-cognitive predictors of health behavior: action self-efficacy and coping self-efficacy. Health Psychol. 2000;19: 487–95. Available: http://www.ncbi.nlm.nih.gov/pubmed/11007157. 11007157

